# Building resilience to early life trauma in belarus and Ukraine

**DOI:** 10.1192/j.eurpsy.2021.211

**Published:** 2021-08-13

**Authors:** O. Kazakova, F. Thomas, O. Suvalo

**Affiliations:** 1 Public Health, Lund University, Malmö, Sweden; 2 Wellcome Centre For Cultures And Environments Of Health, University of Exeter, Exeter, United Kingdom; 3 The Institute Of Mental Health, The Ukrainian Catholic University, Lviv, Ukraine

## Abstract

**Abstract Body:**

Early life trauma (ELT) refers to various types of adversity that occur during the early years (usually defined as the first 5 years) of a person’s life. It is a key determinant of mental health and well-being throughout the life course. A series of three workshops on early life trauma and mental health care were conducted in Belarus and Ukraine in 2018-2019 to support stakeholders and service providers to better understand and respond to ELT, and to support the development of a network of ELT specialists dedicated to finding common goals, pooling cross-disciplinary data and sharing experiences and good practice across countries. The workshops found that different attitudes, expectations and experiences amongst stakeholders and service providers could hinder the development of consistent, effective and empowering care in Belarus and Ukraine. However, opportunities for more protective and health-enhancing responses were also identified, including the need for: evidence-based education and training; clear roles and communication pathways across sectors; and inter-sectoral partnerships and networks to leverage resources, mitigate practitioner burnout, and build a continuum of support within communities. Findings have been disseminated through a directory of resources in Belarus, a project webpage (www.earlylifetrauma.info) and a report on ELT in Belarus and Ukraine published by WHO Europe.

**Disclosure:**

No significant relationships.

